# 1-[9-Ethyl-6-(2-methyl­benzo­yl)-9*H*-carbazol-3-yl]ethanone

**DOI:** 10.1107/S1600536809016080

**Published:** 2009-05-07

**Authors:** Ye-Chao Hang, Jin-Xiu Ji, Min-Dong Chen

**Affiliations:** a College of Environmental Science and Engineering, Nanjing University of Information Science and Technology, Nanjing 210044, People’s Republic of China; b Research & Development Center, Sinochem Jiangsu Corporation, Nanjing 210005, People’s Republic of China

## Abstract

In the title compound, C_24_H_21_NO_2_, the pendant benzene ring is inclined at a dihedral angle of 86.66 (18)° with respect to the adjacent aromatic ring of the carbozole unit. In the crystal structure, symmetry-related mol­ecules are linked *via* C—H⋯O and C—H⋯π inter­actions.

## Related literature

For carbazole-containing compounds used as organic opto-electronic materials, see: Bai *et al.* (2007 ▶); Liu *et al.* (2009 ▶); Promarak *et al.* (2007 ▶). For the synthesis, see: Feng *et al.* (2007 ▶). For bond-length data, see: Allen *et al.* (1987 ▶).
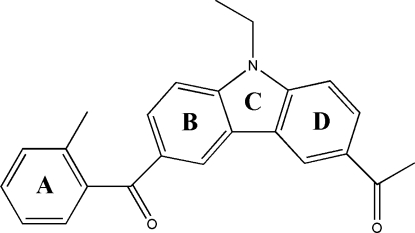


## Experimental

### 

#### Crystal data

C_24_H_21_NO_2_
*M* *_r_* = 355.42
Orthorhombic, 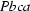

*a* = 13.066 (3) Å
*b* = 13.416 (3) Å
*c* = 21.987 (4) Å
*V* = 3854.2 (13) Å^3^
*Z* = 8
Mo *K*α radiation
μ = 0.08 mm^−1^
*T* = 298 K
0.30 × 0.20 × 0.10 mm

#### Data collection

Enraf–Nonius CAD-4 diffractometer
Absorption correction: ψ scan (North *et al.*, 1968 ▶) *T*_min_ = 0.977, *T*_max_ = 0.992
3492 measured reflections
3492 independent reflections
1733 reflections with *I* > 2σ(*I*)
3 standard reflections every 200 reflections intensity decay: 1%

#### Refinement

*R*[*F* ^2^ > 2σ(*F* ^2^)] = 0.068
*wR*(*F* ^2^) = 0.178
*S* = 1.06
3492 reflections
244 parameters
7 restraints
H-atom parameters constrained
Δρ_max_ = 0.21 e Å^−3^
Δρ_min_ = −0.21 e Å^−3^

### 

Data collection: *CAD-4 Software* (Enraf–Nonius, 1985 ▶); cell refinement: *CAD-4 Software*; data reduction: *XCAD4* (Harms & Wocadlo, 1995 ▶); program(s) used to solve structure: *SHELXS97* (Sheldrick, 2008 ▶); program(s) used to refine structure: *SHELXL97* (Sheldrick, 2008 ▶); molecular graphics: *SHELXTL* (Sheldrick, 2008 ▶); software used to prepare material for publication: *SHELXTL*.

## Supplementary Material

Crystal structure: contains datablocks I, global. DOI: 10.1107/S1600536809016080/su2106sup1.cif

Structure factors: contains datablocks I. DOI: 10.1107/S1600536809016080/su2106Isup2.hkl

Additional supplementary materials:  crystallographic information; 3D view; checkCIF report

## Figures and Tables

**Table 1:** Hydrogen-bond geometry (Å, °)

*D*—H⋯*A*	*D*—H	H⋯*A*	*D*⋯*A*	*D*—H⋯*A*
C11—H11*A*⋯O1^i^	0.93	2.57	3.447 (5)	157
C16—H16*A*⋯O1^i^	0.97	2.54	3.476 (5)	163
C3—H3*A*⋯CgB^ii^	0.93	2.78	3.671 (5)	161

Symmetry codes: (i) 
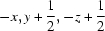
; (ii) 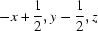. *CgB* is the centroid of the C9–C14 ring.

**Fig. 1.**
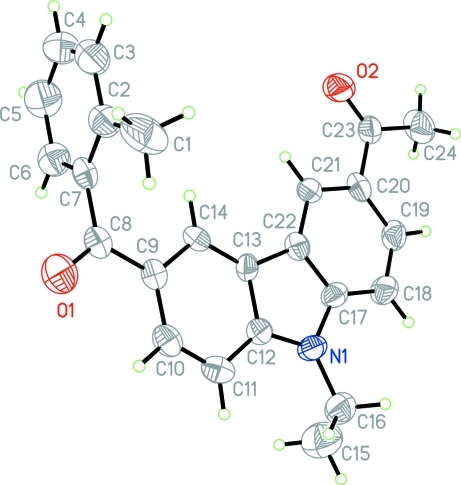
The molecular structure of the title compound, with the atom-numbering scheme. Displacement ellipsoids are drawn at the 40% probability level.

**Fig. 2.**
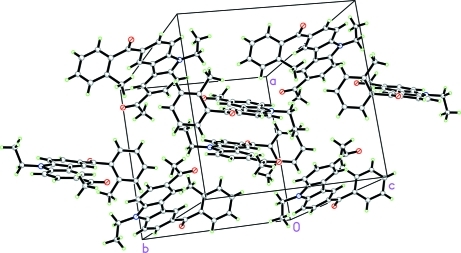
A view along the c axis of the crystal structure of the title compound.

## supplementary crystallographic information

### Comment

The title compound is an important intermediate in the synthesis of carbazole-containing compounds used as organic optoelectronic materials, which have large π—π conjugated networks (Bai *et al.* 2007; Promarak *et al.* 2007; Liu *et al.* 2009). Our interest in this field of research lead us to synthesize, and to report here on the crystal structure of the title compound.

The molecular structure of the title compound is illustrated in Fig. 1. The geometrical parameters are within normal ranges (Allen *et al.*, 1987). The carbozole moiety is slighty bowed with ring B (C9—C14) being inclined to ring D (C17—C22) by 3.04 (19)°. The central ring C (N1,C12,C13,C17,C22) is inclined to rings B and D by 2.76 (18) and 0.30 (18)°, respectively. Ring A (C2—C7) is inclined to ring B by 86.66 (18)°.

In the crystal structure symmetry related molecules are linked *via* C—H···O and C—H···π interactions (Table 1 and Fig. 2).

### Experimental

The title compound was prepared by a slight modification of a method reported in the literature (Feng *et al.*, 2007). That is, the title compound was recrystalized from a mixture of methanol and dichloromethane (V/V = 2:1). On solw evaporation of the solvent colourless block-like crystals appeared after *ca* 4 days.

### Refinement

H atoms were positioned geometrically [C—H = 0.93 - 0.96 Å] and constrained to ride on their parent atoms [*U*_iso_(H) = *xU*_eq_(C), where *x* = 1.2 for aromatic H, and = 1.5 for methyl H].

### Crystal data

**Table:**

C_24_H_21_NO_2_	*F*(000) = 1504
*M**_r_* = 355.42	*D*_x_ = 1.225 Mg m^−^^3^
Orthorhombic, *P**b**c**a*	Mo *K*α radiation, λ = 0.71073 Å
Hall symbol: -P 2ac 2ab	Cell parameters from 25 reflections
*a* = 13.066 (3) Å	θ = 9–13°
*b* = 13.416 (3) Å	µ = 0.08 mm^−^^1^
*c* = 21.987 (4) Å	*T* = 298 K
*V* = 3854.2 (13) Å^3^	Block, colorless
*Z* = 8	0.30 × 0.20 × 0.10 mm

### Data collection

**Table:**

Enraf–Nonius CAD-4 diffractometer	1733 reflections with *I* > 2σ(*I*)
Radiation source: fine-focus sealed tube	*R*_int_ = 0.0000
graphite	θ_max_ = 25.3°, θ_min_ = 1.9°
ω/2θ scans	*h* = 0→15
Absorption correction: ψ scan (North *et al.*, 1968)	*k* = 0→16
*T*_min_ = 0.977, *T*_max_ = 0.992	*l* = 0→26
3492 measured reflections	3 standard reflections every 200 reflections
3492 independent reflections	intensity decay: 1%

### Refinement

**Table:**

Refinement on *F*^2^	Primary atom site location: structure-invariant direct methods
Least-squares matrix: full	Secondary atom site location: difference Fourier map
*R*[*F*^2^ > 2σ(*F*^2^)] = 0.068	Hydrogen site location: inferred from neighbouring sites
*wR*(*F*^2^) = 0.178	H-atom parameters constrained
*S* = 1.06	*w* = 1/[σ^2^(*F*_o_^2^) + (0.07*P*)^2^ + *P*] where *P* = (*F*_o_^2^ + 2*F*_c_^2^)/3
3492 reflections	(Δ/σ)_max_ < 0.001
244 parameters	Δρ_max_ = 0.21 e Å^−^^3^
7 restraints	Δρ_min_ = −0.21 e Å^−^^3^

### Special details

**Table:**

Geometry. All e.s.d.'s (except the e.s.d. in the dihedral angle between two l.s. planes) are estimated using the full covariance matrix. The cell e.s.d.'s are taken into account individually in the estimation of e.s.d.'s in distances, angles and torsion angles; correlations between e.s.d.'s in cell parameters are only used when they are defined by crystal symmetry. An approximate (isotropic) treatment of cell e.s.d.'s is used for estimating e.s.d.'s involving l.s. planes.
Refinement. Refinement of *F*^2^ against ALL reflections. The weighted *R*-factor *wR* and goodness of fit *S* are based on *F*^2^, conventional *R*-factors *R* are based on *F*, with *F* set to zero for negative *F*^2^. The threshold expression of *F*^2^ > σ(*F*^2^) is used only for calculating *R*-factors(gt) *etc*. and is not relevant to the choice of reflections for refinement. *R*-factors based on *F*^2^ are statistically about twice as large as those based on *F*, and *R*- factors based on ALL data will be even larger.

### Fractional atomic coordinates and isotropic or equivalent isotropic displacement parameters (Å^2^)

**Table:**

	*x*	*y*	*z*	*U*_iso_*/*U*_eq_	
N1	0.0687 (2)	0.16753 (19)	0.42892 (12)	0.0504 (8)	
O1	0.0131 (2)	−0.1722 (2)	0.23132 (11)	0.0875 (10)	
O2	0.2544 (2)	−0.1349 (2)	0.61640 (11)	0.0756 (9)	
C1	0.2478 (3)	−0.2480 (4)	0.2692 (2)	0.0959 (15)	
H1A	0.2169	−0.1881	0.2539	0.144*	
H1B	0.2712	−0.2879	0.2357	0.144*	
H1C	0.3047	−0.2312	0.2948	0.144*	
C2	0.1690 (3)	−0.3066 (3)	0.30595 (16)	0.0585 (10)	
C3	0.1953 (3)	−0.3949 (3)	0.33371 (19)	0.0737 (12)	
H3A	0.2614	−0.4197	0.3291	0.088*	
C4	0.1260 (4)	−0.4469 (3)	0.3680 (2)	0.0791 (13)	
H4A	0.1452	−0.5065	0.3862	0.095*	
C5	0.0289 (4)	−0.4116 (3)	0.37538 (19)	0.0795 (13)	
H5A	−0.0184	−0.4462	0.3989	0.095*	
C6	0.0025 (3)	−0.3240 (3)	0.34747 (17)	0.0634 (11)	
H6A	−0.0639	−0.3001	0.3518	0.076*	
C7	0.0713 (3)	−0.2700 (2)	0.31316 (14)	0.0469 (9)	
C8	0.0415 (3)	−0.1750 (3)	0.28459 (15)	0.0506 (9)	
C9	0.0412 (2)	−0.0846 (2)	0.32201 (14)	0.0441 (8)	
C10	0.0086 (3)	0.0068 (3)	0.29808 (14)	0.0530 (9)	
H10A	−0.0153	0.0084	0.2582	0.064*	
C11	0.0103 (3)	0.0925 (3)	0.33004 (15)	0.0525 (9)	
H11A	−0.0160	0.1512	0.3138	0.063*	
C12	0.0532 (2)	0.0903 (2)	0.38852 (14)	0.0435 (8)	
C13	0.0851 (2)	−0.0001 (2)	0.41460 (13)	0.0398 (8)	
C14	0.0809 (2)	−0.0866 (2)	0.38160 (13)	0.0418 (8)	
H14A	0.1041	−0.1461	0.3983	0.050*	
C15	−0.0522 (4)	0.3030 (3)	0.4458 (2)	0.1066 (17)	
H15A	−0.0635	0.3724	0.4377	0.160*	
H15B	−0.0506	0.2922	0.4890	0.160*	
H15C	−0.1067	0.2645	0.4283	0.160*	
C16	0.0474 (3)	0.2716 (3)	0.41870 (18)	0.0673 (11)	
H16A	0.0460	0.2844	0.3753	0.081*	
H16B	0.1020	0.3113	0.4362	0.081*	
C17	0.1146 (3)	0.1274 (2)	0.48129 (15)	0.0462 (8)	
C18	0.1455 (3)	0.1773 (3)	0.53372 (15)	0.0561 (10)	
H18A	0.1379	0.2458	0.5382	0.067*	
C19	0.1877 (3)	0.1190 (3)	0.57813 (16)	0.0545 (10)	
H19A	0.2101	0.1499	0.6136	0.065*	
C20	0.1989 (2)	0.0176 (3)	0.57334 (14)	0.0477 (9)	
C21	0.1685 (2)	−0.0315 (2)	0.52034 (14)	0.0442 (8)	
H21A	0.1769	−0.1000	0.5158	0.053*	
C22	0.1251 (2)	0.0261 (2)	0.47458 (14)	0.0439 (8)	
C23	0.2439 (3)	−0.0457 (3)	0.62258 (16)	0.0537 (9)	
C24	0.2761 (3)	0.0048 (3)	0.68053 (16)	0.0792 (13)	
H24A	0.3027	−0.0439	0.7083	0.119*	
H24B	0.2181	0.0374	0.6985	0.119*	
H24C	0.3281	0.0533	0.6718	0.119*	

### Atomic displacement parameters (Å^2^)

**Table:**

	*U*^11^	*U*^22^	*U*^33^	*U*^12^	*U*^13^	*U*^23^
N1	0.062 (2)	0.0374 (16)	0.0517 (16)	0.0044 (14)	−0.0039 (15)	0.0025 (13)
O1	0.126 (3)	0.0832 (19)	0.0536 (16)	0.0019 (17)	−0.0341 (18)	−0.0082 (14)
O2	0.092 (2)	0.0652 (19)	0.0696 (17)	0.0159 (15)	−0.0320 (16)	−0.0102 (15)
C1	0.055 (3)	0.126 (4)	0.107 (3)	0.009 (3)	0.000 (3)	0.025 (3)
C2	0.057 (3)	0.061 (2)	0.058 (2)	−0.001 (2)	−0.007 (2)	0.0023 (19)
C3	0.063 (3)	0.075 (3)	0.083 (3)	−0.003 (2)	−0.014 (2)	0.008 (2)
C4	0.088 (4)	0.065 (3)	0.084 (3)	0.004 (3)	−0.021 (3)	0.014 (2)
C5	0.088 (4)	0.082 (3)	0.068 (3)	−0.010 (3)	0.000 (3)	0.017 (2)
C6	0.062 (3)	0.066 (3)	0.062 (2)	0.001 (2)	0.001 (2)	−0.005 (2)
C7	0.045 (2)	0.058 (2)	0.0376 (17)	−0.0050 (18)	−0.0094 (16)	−0.0059 (16)
C8	0.047 (2)	0.059 (2)	0.045 (2)	−0.0036 (17)	−0.0077 (17)	−0.0025 (17)
C9	0.040 (2)	0.052 (2)	0.0400 (18)	−0.0051 (16)	−0.0008 (15)	−0.0030 (16)
C10	0.054 (2)	0.067 (2)	0.0377 (17)	−0.0016 (19)	−0.0066 (17)	−0.0007 (18)
C11	0.050 (2)	0.059 (2)	0.048 (2)	0.0083 (18)	−0.0008 (18)	0.0093 (18)
C12	0.039 (2)	0.044 (2)	0.0478 (19)	−0.0004 (16)	−0.0017 (16)	−0.0002 (17)
C13	0.0384 (19)	0.0406 (19)	0.0406 (17)	−0.0100 (15)	−0.0022 (15)	−0.0006 (16)
C14	0.0318 (18)	0.051 (2)	0.0429 (18)	0.0050 (15)	−0.0041 (15)	0.0022 (16)
C15	0.109 (4)	0.082 (3)	0.129 (4)	0.016 (3)	−0.001 (4)	−0.001 (3)
C16	0.082 (3)	0.057 (2)	0.063 (2)	−0.008 (2)	−0.010 (2)	0.0046 (19)
C17	0.049 (2)	0.037 (2)	0.053 (2)	0.0033 (15)	0.0019 (18)	−0.0017 (16)
C18	0.067 (3)	0.045 (2)	0.055 (2)	0.0018 (18)	−0.003 (2)	−0.0111 (18)
C19	0.057 (3)	0.053 (2)	0.054 (2)	−0.0043 (18)	−0.006 (2)	−0.0172 (18)
C20	0.035 (2)	0.064 (3)	0.0443 (18)	0.0001 (17)	−0.0045 (16)	−0.0131 (17)
C21	0.043 (2)	0.0440 (19)	0.0459 (18)	0.0038 (16)	−0.0064 (16)	−0.0024 (15)
C22	0.037 (2)	0.052 (2)	0.0425 (18)	−0.0023 (16)	0.0001 (16)	−0.0041 (15)
C23	0.048 (2)	0.051 (2)	0.062 (2)	−0.0021 (18)	−0.0097 (19)	−0.0116 (18)
C24	0.084 (3)	0.095 (3)	0.059 (2)	0.006 (3)	−0.024 (2)	−0.019 (2)

### Geometric parameters (Å, °)

**Table:**

N1—C12	1.380 (4)	C11—H11A	0.9300
N1—C17	1.406 (4)	C12—C13	1.405 (4)
N1—C16	1.442 (4)	C13—C14	1.369 (4)
O1—C8	1.229 (4)	C13—C22	1.462 (4)
O2—C23	1.212 (4)	C14—H14A	0.9300
C1—C2	1.527 (5)	C15—C16	1.492 (6)
C1—H1A	0.9600	C15—H15A	0.9600
C1—H1B	0.9600	C15—H15B	0.9600
C1—H1C	0.9600	C15—H15C	0.9600
C2—C3	1.376 (5)	C16—H16A	0.9700
C2—C7	1.377 (5)	C16—H16B	0.9700
C3—C4	1.369 (5)	C17—C22	1.373 (4)
C3—H3A	0.9300	C17—C18	1.393 (4)
C4—C5	1.365 (6)	C18—C19	1.367 (5)
C4—H4A	0.9300	C18—H18A	0.9300
C5—C6	1.370 (5)	C19—C20	1.373 (5)
C5—H5A	0.9300	C19—H19A	0.9300
C6—C7	1.379 (5)	C20—C21	1.397 (4)
C6—H6A	0.9300	C20—C23	1.496 (5)
C7—C8	1.473 (5)	C21—C22	1.390 (4)
C8—C9	1.466 (4)	C21—H21A	0.9300
C9—C10	1.399 (4)	C23—C24	1.503 (5)
C9—C14	1.409 (4)	C24—H24A	0.9600
C10—C11	1.348 (5)	C24—H24B	0.9600
C10—H10A	0.9300	C24—H24C	0.9600
C11—C12	1.403 (4)		
			
C12—N1—C17	107.6 (2)	C12—C13—C22	105.4 (3)
C12—N1—C16	126.8 (3)	C13—C14—C9	119.4 (3)
C17—N1—C16	125.5 (3)	C13—C14—H14A	120.3
C2—C1—H1A	109.5	C9—C14—H14A	120.3
C2—C1—H1B	109.5	C16—C15—H15A	109.5
H1A—C1—H1B	109.5	C16—C15—H15B	109.5
C2—C1—H1C	109.5	H15A—C15—H15B	109.5
H1A—C1—H1C	109.5	C16—C15—H15C	109.5
H1B—C1—H1C	109.5	H15A—C15—H15C	109.5
C3—C2—C7	119.2 (4)	H15B—C15—H15C	109.5
C3—C2—C1	120.6 (4)	N1—C16—C15	112.3 (3)
C7—C2—C1	120.1 (3)	N1—C16—H16A	109.1
C4—C3—C2	121.1 (4)	C15—C16—H16A	109.1
C4—C3—H3A	119.4	N1—C16—H16B	109.1
C2—C3—H3A	119.4	C15—C16—H16B	109.1
C5—C4—C3	120.3 (4)	H16A—C16—H16B	107.9
C5—C4—H4A	119.9	C22—C17—C18	122.4 (3)
C3—C4—H4A	119.9	C22—C17—N1	109.5 (3)
C4—C5—C6	118.6 (4)	C18—C17—N1	128.1 (3)
C4—C5—H5A	120.7	C19—C18—C17	115.7 (3)
C6—C5—H5A	120.7	C19—C18—H18A	122.1
C5—C6—C7	122.1 (4)	C17—C18—H18A	122.1
C5—C6—H6A	118.9	C18—C19—C20	123.7 (3)
C7—C6—H6A	118.9	C18—C19—H19A	118.1
C2—C7—C6	118.7 (3)	C20—C19—H19A	118.1
C2—C7—C8	120.3 (3)	C19—C20—C21	120.1 (3)
C6—C7—C8	121.0 (3)	C19—C20—C23	123.3 (3)
O1—C8—C9	120.6 (3)	C21—C20—C23	116.6 (3)
O1—C8—C7	120.8 (3)	C22—C21—C20	117.2 (3)
C9—C8—C7	118.5 (3)	C22—C21—H21A	121.4
C10—C9—C14	118.6 (3)	C20—C21—H21A	121.4
C10—C9—C8	121.0 (3)	C17—C22—C21	120.9 (3)
C14—C9—C8	120.3 (3)	C17—C22—C13	107.4 (3)
C11—C10—C9	123.1 (3)	C21—C22—C13	131.6 (3)
C11—C10—H10A	118.5	O2—C23—C20	121.6 (3)
C9—C10—H10A	118.5	O2—C23—C24	120.6 (4)
C10—C11—C12	117.8 (3)	C20—C23—C24	117.9 (3)
C10—C11—H11A	121.1	C23—C24—H24A	109.5
C12—C11—H11A	121.1	C23—C24—H24B	109.5
N1—C12—C11	129.3 (3)	H24A—C24—H24B	109.5
N1—C12—C13	110.0 (3)	C23—C24—H24C	109.5
C11—C12—C13	120.7 (3)	H24A—C24—H24C	109.5
C14—C13—C12	120.2 (3)	H24B—C24—H24C	109.5
C14—C13—C22	134.2 (3)		
			
C7—C2—C3—C4	0.3 (6)	C12—C13—C14—C9	−2.1 (5)
C1—C2—C3—C4	178.5 (4)	C22—C13—C14—C9	−176.2 (3)
C2—C3—C4—C5	−0.2 (6)	C10—C9—C14—C13	1.1 (5)
C3—C4—C5—C6	0.6 (6)	C8—C9—C14—C13	176.4 (3)
C4—C5—C6—C7	−1.1 (6)	C12—N1—C16—C15	98.8 (4)
C3—C2—C7—C6	−0.8 (5)	C17—N1—C16—C15	−84.8 (4)
C1—C2—C7—C6	−178.9 (3)	C12—N1—C17—C22	−1.7 (4)
C3—C2—C7—C8	179.2 (3)	C16—N1—C17—C22	−178.6 (3)
C1—C2—C7—C8	1.1 (5)	C12—N1—C17—C18	179.1 (3)
C5—C6—C7—C2	1.2 (5)	C16—N1—C17—C18	2.1 (6)
C5—C6—C7—C8	−178.8 (3)	C22—C17—C18—C19	0.4 (5)
C2—C7—C8—O1	84.0 (4)	N1—C17—C18—C19	179.6 (3)
C6—C7—C8—O1	−95.9 (4)	C17—C18—C19—C20	−0.8 (5)
C2—C7—C8—C9	−99.3 (4)	C18—C19—C20—C21	1.4 (5)
C6—C7—C8—C9	80.8 (4)	C18—C19—C20—C23	−179.1 (3)
O1—C8—C9—C10	0.0 (5)	C19—C20—C21—C22	−1.5 (5)
C7—C8—C9—C10	−176.8 (3)	C23—C20—C21—C22	179.0 (3)
O1—C8—C9—C14	−175.3 (3)	C18—C17—C22—C21	−0.5 (5)
C7—C8—C9—C14	8.0 (5)	N1—C17—C22—C21	−179.9 (3)
C14—C9—C10—C11	−2.3 (5)	C18—C17—C22—C13	−180.0 (3)
C8—C9—C10—C11	−177.6 (3)	N1—C17—C22—C13	0.7 (4)
C9—C10—C11—C12	4.4 (5)	C20—C21—C22—C17	1.1 (5)
C17—N1—C12—C11	−179.7 (3)	C20—C21—C22—C13	−179.6 (3)
C16—N1—C12—C11	−2.8 (6)	C14—C13—C22—C17	175.3 (3)
C17—N1—C12—C13	2.0 (4)	C12—C13—C22—C17	0.5 (4)
C16—N1—C12—C13	178.9 (3)	C14—C13—C22—C21	−4.1 (6)
C10—C11—C12—N1	176.5 (3)	C12—C13—C22—C21	−178.8 (3)
C10—C11—C12—C13	−5.3 (5)	C19—C20—C23—O2	−178.7 (4)
N1—C12—C13—C14	−177.2 (3)	C21—C20—C23—O2	0.8 (5)
C11—C12—C13—C14	4.3 (5)	C19—C20—C23—C24	1.4 (5)
N1—C12—C13—C22	−1.6 (4)	C21—C20—C23—C24	−179.2 (3)
C11—C12—C13—C22	179.9 (3)		

### Hydrogen-bond geometry (Å, °)

**Table:**

*D*—H···*A*	*D*—H	H···*A*	*D*···*A*	*D*—H···*A*
C11—H11A···O1^i^	0.93	2.57	3.447 (5)	157
C16—H16A···O1^i^	0.97	2.54	3.476 (5)	163
C3—H3A···CgB^ii^	0.93	2.78	3.671 (5)	161

Symmetry codes: (i) −*x*, *y*+1/2, −*z*+1/2; (ii) −*x*+1/2, *y*−1/2, *z*.
